# Prevalence and risk factors for type 2 diabetes mellitus with Prader–Willi syndrome: a single center experience

**DOI:** 10.1186/s13023-017-0702-5

**Published:** 2017-08-30

**Authors:** Aram Yang, Jinsup Kim, Sung Yoon Cho, Dong-Kyu Jin

**Affiliations:** Department of Pediatics, Samsung Medical Center, Sungkyunkwan University School of Medicine, 81 Irwon-ro, Gangnam-gu, Seoul, 06351 South Korea

**Keywords:** Prader–Willi syndrome, Diabetes mellitus, Insulin, homeostasis model of assessment-insulin, Obesity

## Abstract

**Background:**

Prader–Willi syndrome (PWS) is often related to severe obesity and type-2 diabetes mellitus (T2DM). However, few studies, and none in Korea, have examined prevalence of T2DM and other variables in PWS. The aim of this study was to identify the prevalence and associated risk factors for T2DM in Korean patients with PWS.

**Methods:**

We performed a retrospective cohort study of the 84 PWS patients aged 10 or over (10.3–35.8 years of age) diagnosed with PWS at Samsung Medical Center from 1994 to 2016. We estimated occurrence of T2DM according to age (10–18 years versus >18 years), body mass index (BMI), genotype, history of growth hormone therapy, homeostasis model of assessment-insulin resistance (HOMA-IR), and the presence of dyslipidemia, hypogonadism, or central precocious puberty. Additionally, we investigated cutoff values of risk factors for development of T2DM.

**Results:**

Twenty-nine of a total 211 patients, diagnosed with PWS over the study period, were diagnosed as having T2DM (13.7%, mean age 15.9 ± 3.6 years). In the >18 years group, obesity, HOMA-IR, and presence of dyslipidemia, hypogonadism, or central precocious puberty were associated with the occurrence of T2DM in univariate analysis. In multivariate logistic regression analysis, only obesity (*p* = 0.001) and HOMA-IR (*p* < 0.001) were significant predictive factors for T2DM. Based on the receiver operating a characteristic curve analysis, the cutoff values of HOMA-IR and BMI for predicting T2DM were >2.7 and >28.49 kg/m^2^, respectively. Of the 29 patients, seven had ≥1 microvascular complication, with non-proliferative diabetic retinopathy in 6 of 7 cases. Advanced age and HOMA-IR were positively correlated with diabetic microvascular complications (*p* < 0.05, Spearman correlation coefficient 0.393 and 0.434, respectively).

**Conclusions:**

The prevalence of diabetes in Korean PWS was similar to that in previous results. BMI and HOMA-IR were strong predictive factors for the development of T2DM in PWS. We specifically suggest the regular monitoring of glucose homeostasis parameters through a detailed settlement of ethnically specific cutoff values for BMI and HOMA-IR in PWS to prevent progression of T2DM and diabetic microvascular complications.

**Electronic supplementary material:**

The online version of this article (10.1186/s13023-017-0702-5) contains supplementary material, which is available to authorized users.

## Background

Prader-Willi syndrome (PWS) is a contiguous gene syndrome that results from a lack of the expression of paternal alleles in the PWS region of chromosome 15q11–13 [1]. The clinical manifestations of PWS include hypotonia, early childhood-onset hyperphagia, characteristic facial appearance, hypogonadism, growth hormone deficiency, mild-to-severe mental retardation, and behavioral disturbance [2].

Although patients with PWS show poor feeding and failure to thrive until nine months of age, they tend to be obese after then due to hypothalamic pituitary dysregulation–induced hyperphagia with a lack of satiety. This can lead to severe obesity in childhood [3], which often progressively develops into type-2 diabetes mellitus (T2DM), which is eventually associated with increased morbidity and mortality in PWS. In addition, uncontrolled DM-induced microvascular complications such as diabetic retinopathy, neuropathy, and nephropathy further impair quality of life in PWS.

Obesity and insulin resistance are known to increase the risk of developing T2DM [4]; however, the occurrence of T2DM can be explained by multifactorial mechanisms, and the causal relationship between obesity and diabetes remains unclear. Moreover, obesity itself might not be a decisive factor of diabetes in PWS, considering the low visceral fat distribution and relative low insulin resistance compared to obese individuals without PWS [5]. The etiology of the development of T2DM in PWS has not yet been clarified and thus further research is warranted.

Previous literature places the prevalence of T2DM in PWS at approximately 7–24% [6]. However, there has been no investigation of T2DM associated with PWS in Korea. Against the dramatically increasing incidence of T2DM in the general population, there are few studies available that are related to T2DM in PWS compared to the number of newly updated studies that have been conducted on T2DM in nonsyndromic population. Moreover, the cutoff values of HOMA-IR and BMI for T2DM are differentiated by race and ethnicity; in particular, the cutoff value for BMI in Asia is practically regarded as 23 kg/m^2^. Thus, population and nation-specific studies are essential even in syndromic patients including PWS [7]. In this regard, additional large-scale elaborate investigations are required to identify the precise mechanism of diabetes in PWS besides establishing the primary and secondary prevention of T2DM. The present study estimated the prevalence of T2DM in Korean patients with PWS and attempted to identify the risk factors related to T2DM.

## Methods

### Patients

This study was approved by the institutional review board at Samsung Medical Center (2017–02-144). We reviewed the charts of 211 patients with PWS confirmed via methylation PCR between March 1994 and August 2016 at Samsung Medical Center. Among them, we selected patients aged 10 or older because the youngest patient with T2DM was 10.1 years old, and we compared two groups by dividing subjects into diabetic and non-diabetic groups. We excluded patients with previous bariatric surgery, chronic kidney disease, or no medical records resulted from a lack of clinic visits within the preceding year.

**Fig. 1:**
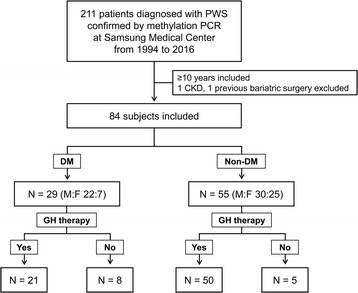
Selection and entry of study subjects in this study

Overall, 84 subjects aged 10.3–35.8 years (i.e., born 1980–2005) were included (Fig. 1). All subjects were Korean individuals with PWS who had visited the hospital at least every six months for a regular checkup. All subjects had homeostasis model assessment-estimated insulin resistance (HOMA-IR) results and body gauge measurement within one year.

Besides, screening for microvascular complications such as comprehensive eye examination by an ophthalmologist, 24-h urine collection with creatinine and microalbumin, and screening for autonomic neuropathy were performed at least annually in PWS patients with T2DM, including at the time of diagnosis.

### Anthropometric and laboratory measurements

We used anthropometric variables measured by same standardized stadiometer only in the pediatric outpatient clinic of the Samsung medical center. Height was measured to the nearest 0.1 cm with a wall-mounted stadiometer (GL-310P, G-tech international, Uijungbu, Korea) and weight was determined to the nearest 0.1 kg on a medical scale by a trained person. Body mass index (BMI) was calculated as weight (kg) divided by height (m) squared. BMI, and height and weight standard deviation scores (SDS) were calculated using the 2007 Korean children and adolescents growth standard [4].

Obesity was defined as the BMI cut-off point >2 SDS in children and adolescent patients (0–18 years) and BMI > 30 in adults [8]. Severe obesity was defined as having a BMI ≥ 120% of the 95th percentile or an absolute BMI ≥ 35 kg/m^2^, whichever was lower based on age and gender [9]. Serum insulin concentrations were measured by immunoradiometric assay using an INS-IRMA kit (BioSource, Nivelles, Belgium).

Peripheral blood samples were obtained after a 12 h overnight fast (at least an eight-hour), and all laboratory data were analyzed at the Samsung medical center. Serum glucose was measured by the hexokinase/glucose-6-phosphate dehydrogenase method. Hemoglobin A1c (HbA1C) expressed as % was measured by high-performance liquid chromatography.

Insulin resistance was measured using HOMA-IR, and calculated as follows: HOMA-IR = fasting insulin (μU/mL) × fasting glucose (mg/dL) /22.5 [10]. The patients were diagnosed with T2DM according to the American Diabetes Association (ADA) criteria as follows [11]: 1) a HbA1c level ≥ 6.5% or 2) a fasting blood sugar (FBS) ≥ 126 mg/Dl (7.0 mmol/L) or 3) a 2-h postprandial plasma blood sugar ≥200 mg/dL during a 75 g oral glucose tolerance test (OGTT) or 4) a random plasma glucose ≥200 mg/dL in a patient with classic symptoms of hyperglycemia or hyperglycemic crisis.

Diabetic nephropathy (DN) is defined by proteinuria >300 mg for 24 h in the setting of diabetes, and microalbuminuria is defined as an albumin excretion of 30–299 mg/24 h.

Diabetic peripheral neuropathy (DPN) was screened for using a Neurometer® Current Perception Threshold (CPT)/C (Neurotron Inc., Denver, CO, USA) in an environment-controlled room. The Neurometer® CPT/C is an electric current generator that provided selective stimulation for three sub-populations of sensory nerve fibers in the skin. The nerve is defined as normal if the CPT is in the normal range, the nerve is defined as in hyperesthesia if the CPT is below its normal range, and the nerve is defined as in hypoesthesia if the CPT is higher than the normal range. The methods of diabetic cardiac autonomic neuropathy assessment include the assessment of symptoms, signs, and cardiovascular autonomic reflex tests based on heart rate and BP variation to deep breathing, Valsalva maneuver, and postural change using the DICAN® evaluation system (Medicore Co., Ltd., Seoul, Korea). DPN was defined as when abnormalities were found in both the CPT and autonomic function test.

### Statistical analysis

The baseline characteristics were described using percentages for categorical variables and means ± SD or mean changes (SD) for continuous variables. Comparisons between diabetic and non-diabetic groups were performed using the Wilcoxon–Mann–Whitney test for categorical variables and Student’s t test for continuous variables. Univariate logistic regression analysis was employed to evaluate the crude effects of the variables on the development of T2DM. Variables associated (*p* < 0.10) with outcomes in the univariate analysis were subject to a multivariate backward stepwise logistic regression analysis to estimate their adjusted effects on the development of T2DM. The odds ratio (OR) and the 95% confidence intervals (CIs) were computed from the regression analyses.

The impact of risk factors for T2DM were evaluated using areas under the curve for sensitivity and specificity, which were constructed (receiver–operator characteristic [ROC] curve [12]) using MedCalc version 17.4 (MedCalc Software, Ostend, Belgium). The ROC curve is a graphical representation of the relationship between sensitivity and specificity based on various anthropometric cut-off values. A perfect test will have an AUC of 1.0, and AUC = 0.5 means that the test performs no better than chance. The optimal cut-off point was identified based on the maximal Youden index (sensitivity + specificity −1).

Spearman rank order correlation was performed to evaluate possible correlations between microvascular complications and independent variables. All statistical analyses were performed using SPSS 23 (IBM Corporation, USA). A *p* value of <0.05 was considered statistically significant.

## Results

The baseline characteristics of all subjects above 10 years old with PWS are shown in Table 1. Out of the 211 diagnostically confirmed PWS patients, T2DM was found in 29 (13.7%). The mean age at DM diagnosis was 15.9 years (10.1–27.0). The diabetic group was 0.4 ± 5.7 years older than the non-diabetic group (15.8 ± 4.1 years). Although the percentage of males in the diabetic group (75.9%), was slightly higher than the non-diabetic group (54.5%), no significant sex ratio distribution was evident. The mean BMI in the diabetic group was 35.7 ± 9.2 kg/m^2^, corresponding to 2.8 ± 1.0 SD, higher than the non-diabetic group (28.3 ± 8.9 kg/m^2^). Twenty-three patients among the 29 (79.3%) in the diabetic group were obese, which was higher than the non-diabetic group (22/55, 40%). The mean HOMA-IR was 6.3 ± 3.5 in the diabetic group, which was higher than in the non-diabetic group (3.4 ± 3.8). Twenty-one (72.4%) patients in the diabetic group and 50 patients (90.9%) in the non-diabetic group had a history of GH treatment. However, the duration of GH treatment prior to the occurrence of T2DM was not significantly different between the diabetic and non-diabetes groups (*p* = 0.140). Meanwhile, the percentage of patients with dyslipidemia taking medications and the patients with hypogonadism who underwent hormone replacement therapy were higher than in the non-diabetic group, at 55.2% and 65.5%, respectively (vs. 20.0% and 36.4% in the non-diabetic group). The proportion of patients with severe obesity was higher in PWS-DM group compared to the 84 subjects in total (*p* < 0.03) (Additional file 1: Fig. S1).

**Table 1:** Baseline characteristics of the patients with PWS

	DM, n (%) 29 (34.5)	Non-DM, n (%) 55 (65.5)	Total, n (%) 84 (100)	*p*-value^*^
Age (years) (range)	20.4 ± 5.7 (12.7–35.8)	15.8 ± 4.1 (10.3–31.8)	17.4 ± 5.1 (10.3–35.8)	< 0.001
Age at diagnosis with DM (years)	15.9 ± 3.6 (10.1–27.0)	-	-	-
Age at diagnosis with PWS (years)	6.9 ± 7.2	4.7 ± 5.0	5.5 ± 5.9	0.151
Gender (male), n (%)	22 (75.9)	30 (54.5)	52 (61.9)	0.057
Genotype (deletion), n (%)	21 (72.4)	38 (69.1)	59 (70.2)	0.757
BMI, kg/m2 (SD)	35.7 ± 9.2 (2.8 ± 1.0)	28.3 ± 8.9 (1.7 ± 1.3)	30.8 ± 9.6 (2.1 ± 1.3)	< 0.001
Obesity^a^, n (%)	23 (79.3)	22 (40)	57 (67.9)	0.001
HOMA-IR	6.3 ± 3.5	3.4 ± 3.8	4.4 ± 3.9	0.001
HbA1C, % (mmol/mol)	8.4 ± 2.3	5.6 ± 0.3	6.6 ± 1.9	< 0.001
Basal insulin (μIU/mL)	45.7 ± 31.1	25.3 ± 23.0	32.5 ± 27.7	0.001
Basal C-peptide (ng/mL)	7.0 ± 2.8	4.8 ± 2.9	5.7 ± 3.0	0.004
Age at GHT start (years)	7.0 ± 3.8	6.7 ± 4.3	6.8 ± 4.1	0.795
Previous GHT duration (years)	6.3 ± 3.3	7.5 ± 3.1	7.2 ± 3.2	0.140
GHT, n (%)	21 (72.4)	50 (90.9)	71 (84.5)	0.027
Dyslipidemia, n (%)	16 (55.2)	11 (20.0)	27 (32.1)	0.001
Hypothyroidism, n (%)	0 (0)	2 (3.6)	2 (2.4)	0.988
Hypogonadism, n (%)	19 (65.5)	20 (36.4)	39 (46.4)	0.011
Central precocious puberty, n (%)	2 (6.9)	12 (21.8)	14 (16.7)	0.071

Data are presented as number (%) or mean ± SD

*DM* Diabetes Mellitus, *DMT* treatment of DM, *GHT* growth hormone treatment, *BMI* body mass index, *HOMA-IR* homeostasis model assessment, *HbA1C* hemoglobin A1C

^*^Significant at *p* < 0.05

^a^In this study, obesity is defined as BMI cut-off point >30 in adults, >2 SDS in children and adolescents

Regression analysis for the prevalence of T2DM was as shown in Table 2. Age above 18 years, obesity, HOMA-IR, dyslipidemia, and hypogonadism were significant risk factors related with T2DM. Growth hormone treatment (GHT) was not a leading cause for the development of T2DM regardless of both age at GHT start and treatment period. In addition, neither genotype nor gender was associated with the development of T2DM. In multivariate analysis after covariation of factors (age, gender, BMI, HOMA-IR), HOMA-IR (OR 1.73, 95% CI 1.31–2.29) and obesity (OR 6.76, 95% CI 2.10–21.70) were significant predictors of T2DM development.

**Table 2:** Univariate and multivariate logistic regression analyses of variables associated with risk of T2DM in PWS

Variables	Univariate analysis		Multivariate analysis	
	OR (95% CI)	*P*-value^*^	OR (95% CI)	*P*-value^*^
Age (>18 years)	6.81 (2.51–18.47)	<.001	1.87 (0.47–7.47)	0.373
Gender (male)	0.38 (0.14–1.04)	0.060	0.60 (0.16–2.28)	0.455
Genotype (deletion)	0.85 (0.30–2.40)	0.756	-	-
Obesity^a^ (BMI > 2SD or >30 kg/m^2^)	5.75 (2.02–16.40)	0.001	6.76 (2.10–21.70)	0.001
HOMA-IR	1.31 (1.09–1.59)	0.005	1.73 (1.31–2.29)	<.001
Dyslipidemia (yes)	4.92 (1.84–13.20)	0.002	1.55 (0.38–6.33)	0.545
GHT (yes)	0.39 (0.11–1.37)	0.141	-	-
Age at GHT start (years)	1.02 (0.90–1.15)	0.792	-	-
Duration of GHT (≥7.2 years)	0.75 (0.27–2.07)	0.572	-	-
Hypogonadism (yes)	3.33 (1.30–8.53)	0.012	2.15 (0.61–7.63)	0.237
Central precocious puberty (yes)	0.27 (0.06–1.28)	0.10	-	-

Variables with *p* < 0.10 were included in a multivariate logistic regression with conditional backward selection model

Abbreviations: *T2DM* type 2 Diabetes Mellitus, *PWS* Prader-Willi syndrome, *OR* odds ratio, *CI* confidence interval, *GHT* growth hormone treatment, *HOMA-IR* homeostasis model assessment, *BMI* body mass index

^*^Significant association was classified as *p* < 0.05

^a^Obesity is defined as BMI cut-off point >30 in adults, >2 SDS in children and adolescents

**Fig. 2:**
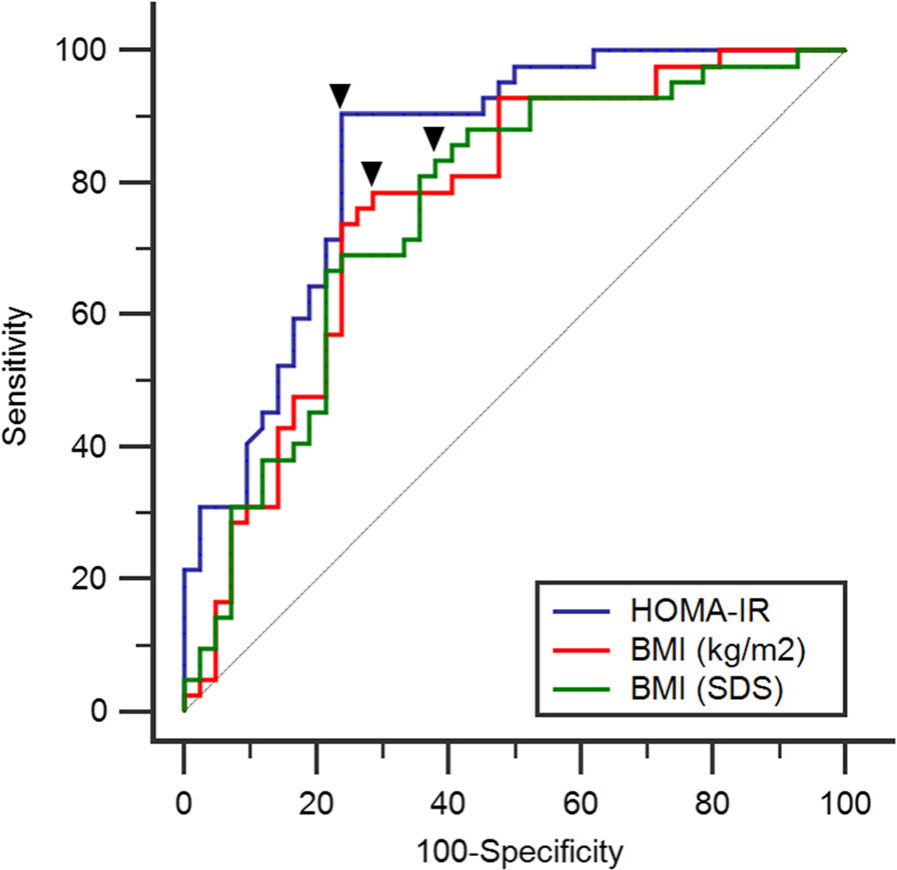
Receiver–Operator Characteristic (ROC) curves predicting the impact of HOMA-IR (blue line), BMI (SDS) (green line), and BMI (kg/m^2^) (red line) on T2DM in PWS. The black line represents the zero discrimination line. Each black triangle denotes the point of cut-off value

Figure 2 shows the ROC curves for detecting T2DM using HOMA-IR, BMI (kg/m^2^), and BMI (SDS). The areas under the ROC curves were 0.843 (95% CI: 0.758–0.927), 0.765 (95% CI: 0.660–0.851), and 0.757 (95% CI: 0.652–0.844), respectively (*p* < 0.0001). HOMA-IR and BMI (SDS and kg/m^2^) were similar, but HOMA-IR was slightly better than BMI at detecting T2DM. The cut-off values for anthropometric indices determined using ROC analysis are summarized in Table 3. The probability of T2DM was increased for HOMA-IR results >2.7 (Youden’s index 0.67), BMI (kg/m^2^) result >28.49 (Youden’s index 0.50), and BMI (SDS) result >1.73 (Youden’s index 0.45). These results were found to be suitable cut-off points for the detection of T2DM, as they possess the highest Youden’s index.

**Table 3:** Cut-off values for HOMA-IR, BMI (kg/m^2^), and BMI (SDS) as predictive values of T2DM development in PWS

	Cut off point	Area under the curve (95% CI)	Sensitivity (95% CI)	Specificity (95% CI)
HOMA-IR	2.7^*^	0.843	90.48	76.19
BMI (kg/m^2^)	28.49^*^	0.765	78.57	71.43
BMI (SDS)	1.73^*^	0.757	83.33	61.90

Abbreviations: *T2DM* type 2 Diabetes Mellitus, *PWS* Prader-Willi syndrome, *HOMA-IR* homeostasis model assessment, *BMI* body mass index, *CI* confidence interval, *SDS* standard deviation score

^*^Significant association was classified as *p* < 0.0001

Among the 29 patients with T2DM, seven showed microvascular complications. Spearman correlation analysis presented that the prevalence of microvascular complications for the occurrence of T2DM was positively associated with aging (r  =  0.393, *p*  =  0.047) and HOMA-IR (r  =  0.434, *p* = 0.027). Meanwhile, the period of having T2DM tended to have a positive association with microvascular complications, but not to a significant degree (r  =  0.370, *p*  =  0.063) (Table 4).

**Table 4:** Correlation analysis for microvascular complications and other variables in the diabetic group of PWS patients

Variables	Correlation coefficient	
	r	*p*-value^*^
Age (years)	0.393	0.047
Age at diagnosis with PWS (years)	0.226	0.267
Age at diagnosis with T2DM (years)	0.104	0.613
HbA1C (%)	0.232	0.254
BMI, kg/m2 (SD)	0.133	0.517
HOMA-IR	0.434	0.027
Age at GHT start (years)	0.189	0.439
Previous GHT duration (years)	0.376	0.058
DM treatment duration (years)	0.370	0.063
From PWS diagnosis to DM diagnosis (years)	−0.156	0.446

Abbreviations: *r* Spearman correlation coefficient, *T2DM* type 2 Diabetes Mellitus, *PWS* Prader-Willi syndrome, *HbA1C* hemoglobin A1C, *GHT* growth hormone treatment, *HOMA-IR* homeostasis model assessment, *BMI* body mass index

^*^Significant association was classified as *p* < 0.05

Out of the seven patients with microvascular complications, nonproliferative retinopathy was found in five (17.2%), microalbuminuria and DPN were shown in two (6.9%) patients, and only one patient showed overt proteinuria (3.4%) (Table 5). The time from the detection of T2DM to these complications was different for each. One patient already had all three microvascular complications at the time of T2DM diagnosis. Notably, HbA1C upon the diagnosis of complications was higher than upon the diagnosis of T2DM. The BMI and HOMA-IR of seven patients with complications showed a tendency to be higher than those of other patients without complications.

**Table 5:** Clinical and auxological data of seven PWS patients with diabetic microvascular complication

Patient no.	Sex	Genotype	Diagnosis age (yr)		Age at Cx onset (yr)	Duration until Cx onset (yr)	BMI, kg/m^2^ at Dx with DM	BMI, kg/m^2^ at Dx with Cx	A1C (%) at Dx with DM	A1C (%) at Dx with Cx	HOMA-IR	Duration of GHT	Duration of DM	Type of DMT	Type of Cx
			PWS	DM											
5	M	Del	10.1	15.0	23.5	8.5	47.55	38.25	7.3	14.0	7.7	NA	13.3	M- > M + S- > R- > N + M + S- > M + S- > M	DR
14	M	Del	1.8	13.8	15.0	1.2	22.53	25.28	9.6	7.2	8.5	3	2.0	M + B- > M	DR
75	M	Del	0.2	17.8	23.0	5.2	41.82	40.48	9.4	9.7	7.9	1.2	5.5	M + S- > M + S + B	DR
12	F	Del	4.8	14.7	19.0	4.3	30.48	29.59	6.9	11.3	10.2	7.5	5.0	M- > M + S- > M + S + B- > M + B + G	DR
44	F	Del	8.0	18.5	22.5	4.0	32.51	31.47	8.7	6.7	7.0	1.8	4.8	M	DN (Mi), DPN
55	F	Del	13.3	14.2	24.2	10.0	51.40	55.46	6.7	12.0	9.5	NA	11.4	M > M + S- > M + S + α- > M + S + α + G- > B + R + M	DN (P)
77	F	Del	27.0	27.0	27.0	0.0	40.20	40.20	12.1	12.1	7.6	NA	5.7	M + S + B	DN (Mi), DR, DPN

Abbreviations: *PWS* Prader-Willi syndrome, *DM* Diabetes Mellitus, *Del* deletion, *Cx* microvascular complication, *A1C* hemoglobin A1C, *HOMA-IR* homeostasis model assessment, *GHT* Growth hormone treatment, *DMT* Treatment of diabetes mellitus, *DN* Diabetic nephropathy, *DR* Non-proliferative diabetic retinopathy, *DPN* Diabetic peripheral neuropathy, *I* insulin, *PO* Oral hypoglycemic drug

## Discussion

This is the first study about the prevalence and risk factors of T2DM in Korean PWS patients to our knowledge. The prevalence of T2DM in the present study was 13.7% (29/211). This proportion is similar to that of previous studies (7–25%) [13–16]. Considering the prevalence of T2DM in adults (6.1–6.9%) [17] and 0.2% in children younger than 18 years in Korea [18], the prevalence rate of T2DM in Korean PWS is sufficiently high to warrant attention.

A recent Italian cohort study revealed a 13.5% prevalence of T2DM, which was similar to our results [15]. This proportion is relatively lower than the results of a Japanese study, which were 26.2% [16], but higher than in a French study, 0% [19]. The differences in the described frequencies of T2DM have presumably resulted from various sizes of the PWS base population, the diagnostic approach to T2DM, the wide range of the age group, and the study period.

Insulin resistance and obesity are well known risk factors in T2DM. However, this relationship has been questioned in PWS due to relative hypoinsulinemia of PWS compared to non-syndromic obese individuals [20–22]. It seems that the reason for this considerably high insulin sensitivity is subcutaneous fat–dominant obesity with reduced visceral fat, higher plasma ghrelin and adiponectin levels, reduced β-cell response to glucose stimulation, and insufficient growth hormone [23–26]. Acylated ghrelin is an orexigenic hormone that is associated with hyperphagia-induced obesity inducing positive energy balance and possibly involved in the development of diabetes in PWS [27]. However, although previous studies have not independently investigated T2DM, a recent study conducted in Italy [15] also showed that HOMA-IR and BMI were independent risk factors associated with T2DM in PWS, supporting our study result. Meanwhile, unlike the Italian results indicating that being >18 years of age was a significant risk factor for altered glucose metabolism (AGM), our result only indicated such significance in univariate analysis, not in multivariate analysis. This result can be explained by the distinction between study populations; 54 out of the 67 patients with AGM in the Italian study were over 18 years old and 52 were obese. In addition, it might have resulted from the difference in research targets, such as studying IFG and IGT, not only T2DM in the Italian study. Considering that age is generally a critical factor for the development of T2DM, the relatively younger age of our study group may be reflected in this outcome. In addition, it is noteworthy that the hypogonadism ratio, is significantly higher in the diabetic group than the non-diabetic group, in our study. Although obesity has been suggested as the important factor in decreased levels of total and free testosterone in T2DM, so far the relationship between hypogonadism and T2DM in PWS has not been clarified due to disease rarity and possibilities of multiple confounding factors [28]. However, our study results demonstrate a more potent approach to the relevance of diabetes and hypogonadism and further researches are needed. The present study also demonstrates that HOMA-IR and BMI have high sensitivity and specificity for assessing risk of T2DM with drawing cut off values through ROC curve analysis. The cutoff value of HOMA-IR of 2.7 for PWS with T2DM in our study is slightly higher than the cutoff value of 2.2–2.5 for metabolic syndrome in the general non-diabetic Korean population [29] and the cutoff value of 2.5 in the general non-diabetic Japanese population [30] but lower than the cutoff value of 3.16 for insulin resistance in Turkish children and adolescents without T2DM [31]. Meanwhile, compared to the cut off value of 4.0 for the diabetic group in Iranian patients [32], our result was considerably lower. While exact comparisons are difficult, the difference between these results suggests the possibility that patients with PWS are less resistant to insulin than non-syndromic patients with T2DM, which support previous research results that show quite high insulin sensitivity [33].

The cutoff value for BMI of 28.49 kg/m^2^ in our study was lower than the cutoff value of 30 kg/m^2^ in the general Caucasian population, but it was higher than the cutoff value of less than 25 kg/m^2^ in Asian population [7, 34, 35]. Our patients were all domestic Korean, which implies that PWS patients might be less likely to develop T2DM compared to non-syndromic population with similar BMI within the same race, which also lends credence to previous studies [5]. However, we need to take account of this influence on the cut off values by considering the differences in characteristics, sample size, study method, and gender in addition to ethnic and racial differences. Although there are no comparative studies on the sensitivity and specificity of the cutoff value for T2DM in PWS, these research attempts suggest that if precise large-scale research is conducted at a national level, it will be possible to increase the early diagnostic rate of T2DM in PWS and to take active measures for its management.

The causal relationship between GH therapy and the occurrence of T2DM has been a controversial issue for a long time. Recent studies have reached the conclusion that fasting insulin levels during GH therapy might be slightly elevated in children with PWS, but this is transient and does not eventually progress to diabetes [15, 36–39]. Furthermore, it is carefully suggested that if growth hormone is used at a low dose under strict metabolic control, it can increase muscle mass and insulin sensitivity while cutting down visceral fat and preventing the development of T2DM [40–42]. Still, there is no direct consensus for treatment target and dosage for central obesity to cut metabolic clusters. Moreover, given our study results, extreme obesity with a cut-off BMI value exceeding 26–28 kg/m^2^ facilitates insulin secretion leading to overt diabetes via insulin resistance regardless of GH therapy, despite the effect of PWS [37, 43, 44].

In our study, age and HOMA-IR were found to be related to the occurrence of microvascular complications, but this study has limitations because it did not have a large number of subjects and was retrospective rather than a long-term prospective study. Several risk factors of microvascular complications have been reported in adults with T2DM, such as duration of diabetes, age, blood pressure, fasting plasma glucose, urinary albumin excretion levels, and elevated C-Reactive Protein levels [45–47]. However, it is difficult to compare our results because there have been few reports of complications related to T2DM in PWS [48]. The disease’s rarity has meant that little research has been conducted despite the high prevalence of T2DM in PWS. Although not yet clear, ethnicity and familial insulin resistance characteristics may be associated with the development of diabetes in PWS, and further studies are expected to examine this.

Another limitations of this study are as follows: 1) The possibility that selection bias occurred in patients who have relatively good compliance with regular hospital follow-ups. 2) The possibility that the average age of study subjects might be lowered due to patients older than 30 years usually having poor compliance, thus being excluded from our study. 3) Failure to perform subcutaneous fat tissue comparison with dual-energy X-ray absorptiometry. 4) The 12-h fasting (at least 8-h) laboratory test was not performed perfectly due to the nature of PWS of intolerance to permanent hunger.

Nevertheless, the present study is worthwhile as the first in Korea on PWS patients diagnosed with T2DM. In addition, anthropometric measurements using the same method and stadiometer minimized measurement error, and a biochemical study conducted in the same laboratory made our results more reliable.

Several studies have reported that insulin resistance would not be significantly involved in the causes of T2DM in PWS due to the better insulin sensitivity of patients compared to obese individuals with PWS [5, 33]. However, according to recent studies including our results, although patients with PWS may have relative insulin sensitivity, insulin resistance and obesity are considered the most important factors in the occurrence of T2DM through the destruction of β-cell function [15, 16, 37].

Our results support that obesity plays a major role in metabolic clusters in both children and adolescents with PWS. Therefore, it is necessary to actively control body weight through dietary control and exercise from early childhood to prevent severe obesity and T2DM.

## Conclusions

In conclusion, the prevalence of T2DM in Korean PWS in our study was similar to the results of previous studies. BMI and HOMA-IR are strong predictive factors for the development of T2DM in PWS. In addition, our results suggest a relatively high cutoff level for the development of T2DM in PWS compared to non-syndromic obese control within the same race.

We emphasize early intervention to prevent severe obesity and the regular monitoring of glucose homeostasis parameters to predict the occurrence of T2DM in PWS. Further longitudinal studies are required to better understand the endocrine and metabolic factors that can determine T2DM development in PWS individuals.

## Additional file

Additional file 1: Figure S1. Stratification of study population according to severity of obesity. Overweight: BMI cutoff point from 1.4 to 2 SDS in children and adolescents patients (0–18 yrs) and BMI from 25 to 30 kg/m^2^ in adults; Obesity: BMI cut-off point >2 SDS (0–18 years) and BMI > 30 kg/m^2^ in adults; Severe obesity: BMI ≥ 120% of the 95th percentile or an absolute BMI ≥ 35 kg/m^2^ (TIFF 362 kb)

## Abbreviations

AGM: Altered glucose metabolism
BMI: Body mass index
CIs: Confidence intervals
CPT: Current Perception Threshold
DN: Diabetic nephropathy
DPN: Diabetic peripheral neuropathy
GH: Growth hormone
GHT: Growth hormone treatment
HbA1C: Hemoglobin A1c
HOMA-IR: Homeostasis model assessment-estimated insulin resistance
OR: Odds ratio
PWS: Prader-Willi syndrome
ROC: Receiver-operator characteristic
SD: Standard deviation
SDS: Standard deviation score
T2DM: Type 2 diabetes mellitus
