# Supplementary figures and images for: Prevalence and risk factors for type 2 diabetes mellitus with Prader–Willi syndrome: a single center experience
Source: Orphanet J Rare Dis. 2017 Aug 30;12:146. doi: 10.1186/s13023-017-0702-5 (PMC5577752; doi:10.1186/s13023-017-0702-5)

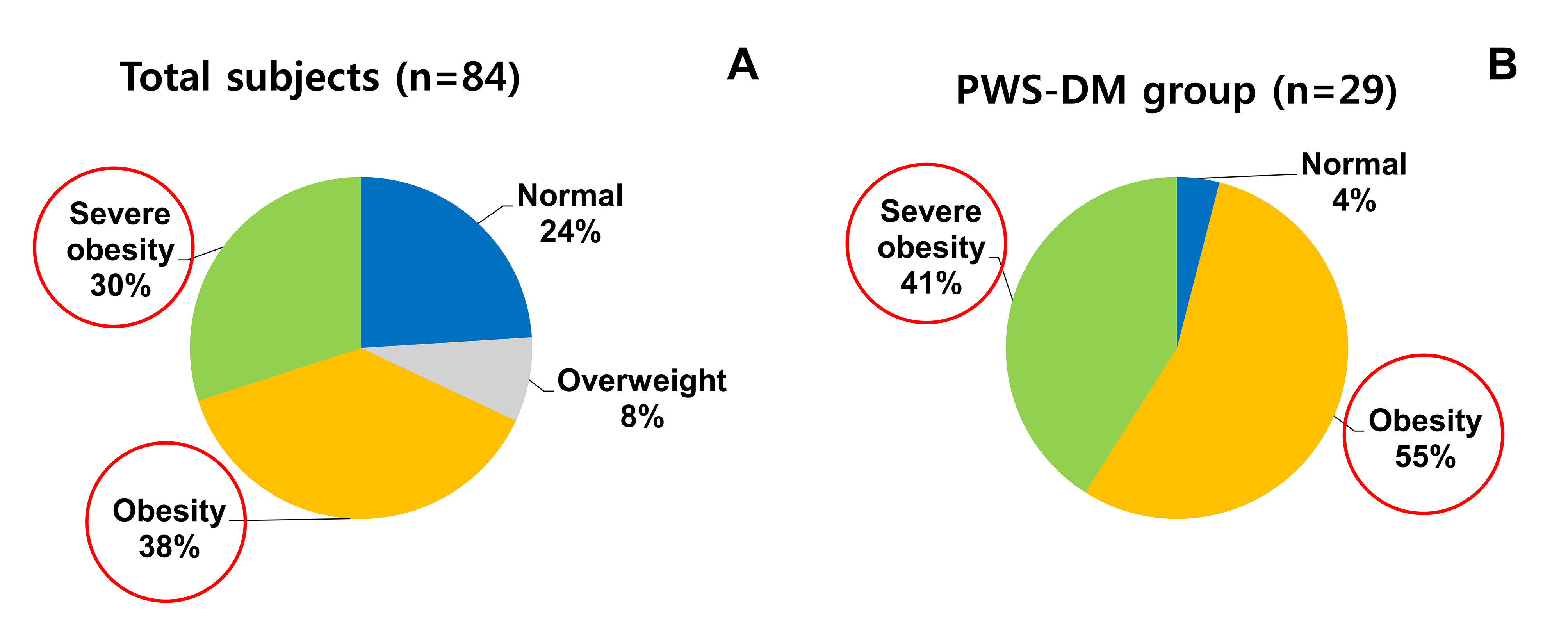

Supplement: Additional file 1: Figure S1. — Stratification of study population according to severity of obesity. Overweight: BMI cutoff point from 1.4 to 2 SDS in children and adolescents patients (0–18 yrs) and BMI from 25 to 30 kg/m2 in adults; Obesity: BMI cut-off point >2 SDS (0–18 years) and BMI > 30 kg/m2 in adults; Severe obesity: BMI ≥ 120% of the 95th percentile or an absolute BMI ≥ 35 kg/m2 (TIFF 362 kb) [file 13023_2017_702_MOESM1_ESM.tif]
